# Moving forward monitoring of the social determinants of health in a country: lessons from England 5 years after the Marmot Review

**DOI:** 10.3402/gha.v9.29627

**Published:** 2016-02-25

**Authors:** Peter O. Goldblatt

**Affiliations:** Department of Epidemiology and Public Health, UCL Institute of Health Equity, London, United Kingdom

**Keywords:** health inequalities, social determinants of health, social gradient, monitoring framework, disaggregation of data

## Abstract

**Background:**

England has a long history of government-commissioned reviews of national inequalities. The latest review, the Marmot Review, was commissioned by a government headed by the same party (the Labour Party) that had introduced the National Health Service in 1948, but the review was implemented by a coalition of different parties (Conservatives and Liberal Democrats). At the same time, a government reform of health services took place, and the monitoring of the existing inequality strategy was changed.

**Objectives:**

This paper examines the lessons that can be learned about indicators for monitoring social determinants of health inequalities from the Marmot Review and recent health inequality strategies in England.

**Design:**

The paper provides a narrative review of key findings on the collection, presentation, and analysis of routine data in England in the past 5 years, comparing what has been learned from the Marmot Review and other evaluations of the first health inequality strategy in England.

**Results:**

The emphasis on monitoring has progressively shifted from monitoring a small number of targets and supporting information to frameworks that monitor across a wide range of determinants of both the causes of ill-health and of health service performance. As these frameworks become ever larger, some consideration is being given to the key indicators.

**Conclusions:**

Although the frameworks used in England for monitoring health inequality strategies have developed considerably since the first strategy began, lessons continue to be learned about how monitoring could be improved. Many of these are applicable to countries initiating or reviewing their strategies.

## Introduction

In November 2008, the Secretary of State for Health in England asked Professor Sir Michael Marmot to chair an independent review to propose the most effective evidence-based strategies for reducing health inequalities in England from 2010 (1). This request followed the publication of the report of the World Health Organization (WHO) Commission on Social Determinants of Health (CSDH) in August 2008 under Sir Michael's chairmanship (2). It also built on three previous reviews in England. Two of these were published in a period when the government's focus was directed away from policies to reduce health inequalities – The Black Report in 1980 (3) and The *Health Divide* in 1987 (4). By contrast, the Acheson Review in 1998 (5) was commissioned by the then-incoming government, which used it to develop the first health inequality strategy in England. This strategy included targets for life expectancy and infant mortality, to be achieved by 2010. These targets were agreed as part of the Public Service Agreement in 2002 (6) and a programme for action published in 2003 (7). Mindful of the English policy history in tackling health inequalities, this paper analyses and summarises lessons learnt for monitoring from the publication of the Marmot Review report, *Fair Society, Healthy Lives* (1), and concurrent policy developments.

## Marmot Review findings and national policy developments

*Fair Society, Healthy Lives* was published in February 2010 (1) shortly after publication of the evaluation of the first national strategy in 2009 (8). The report made a number of recommendations, central to which was that reducing health inequalities required action on six policy objectives:

- Give every child the best start in life
- Enable all children, young people, and adults to maximise their capabilities and have control over their lives
- Create fair employment and good work for all
- Ensure a healthy standard of living for all
- Create and develop healthy and sustainable places and communities
- Strengthen the role and impact of ill health prevention

Drawing on the earlier reviews [see commentary by Bambra et al. (9)], the report emphasised that delivering these policy objectives would require action by central and local government, the National Health Service (NHS), the private sector, and community groups.

Shortly after publication, there was again a change in government and it fell to the new government to respond to these recommendations. This response was contained in the government white paper *Healthy Lives, Healthy People: Our strategy for Public Health in England* (10). This stated that the government's strategy ‘adopts its [*Fair Society, Healthy Lives*] life course framework for tackling the wider social determinants of health (SDH). The new approach will aim to build people's self-esteem, confidence and resilience right from infancy – with stronger support for early years’.

To support this new approach the government reformed the public health system, devolving to local levels wherever possible. Public health was transferred from the health service to local government, setting the following aims:

- Strengthening self-esteem, confidence, and personal responsibility
- Positively promoting ‘healthier’ behaviours and lifestyles
- Adapting the environment to make healthy choices easier

The government established a new national body, Public Health England, funding services as follows:

- Granting the public health ring-fenced budget to local government
- Asking the NHS to commission services, such as screening services, and the relevant elements of general practice contracts
- Commissioning or providing services directly, for example national purchasing of vaccines, national communication campaigns, or health protection functions

Local authorities were encouraged to incorporate the approaches set out in *Fair Society, Healthy Lives* in their health and well-being strategies. By the end of the first year, the local public health response was mixed. Around three-quarters had made some mention of the ‘Marmot principles’ in their strategies, although fewer had adopted an approach that was fully engaged with the social determinants agenda and the proposed monitoring framework.

## Monitoring of inequalities in health and its social determinants: Marmot Review proposals and subsequent developments

### Marmot Review and the inequality strategy

**Fig. 1:**
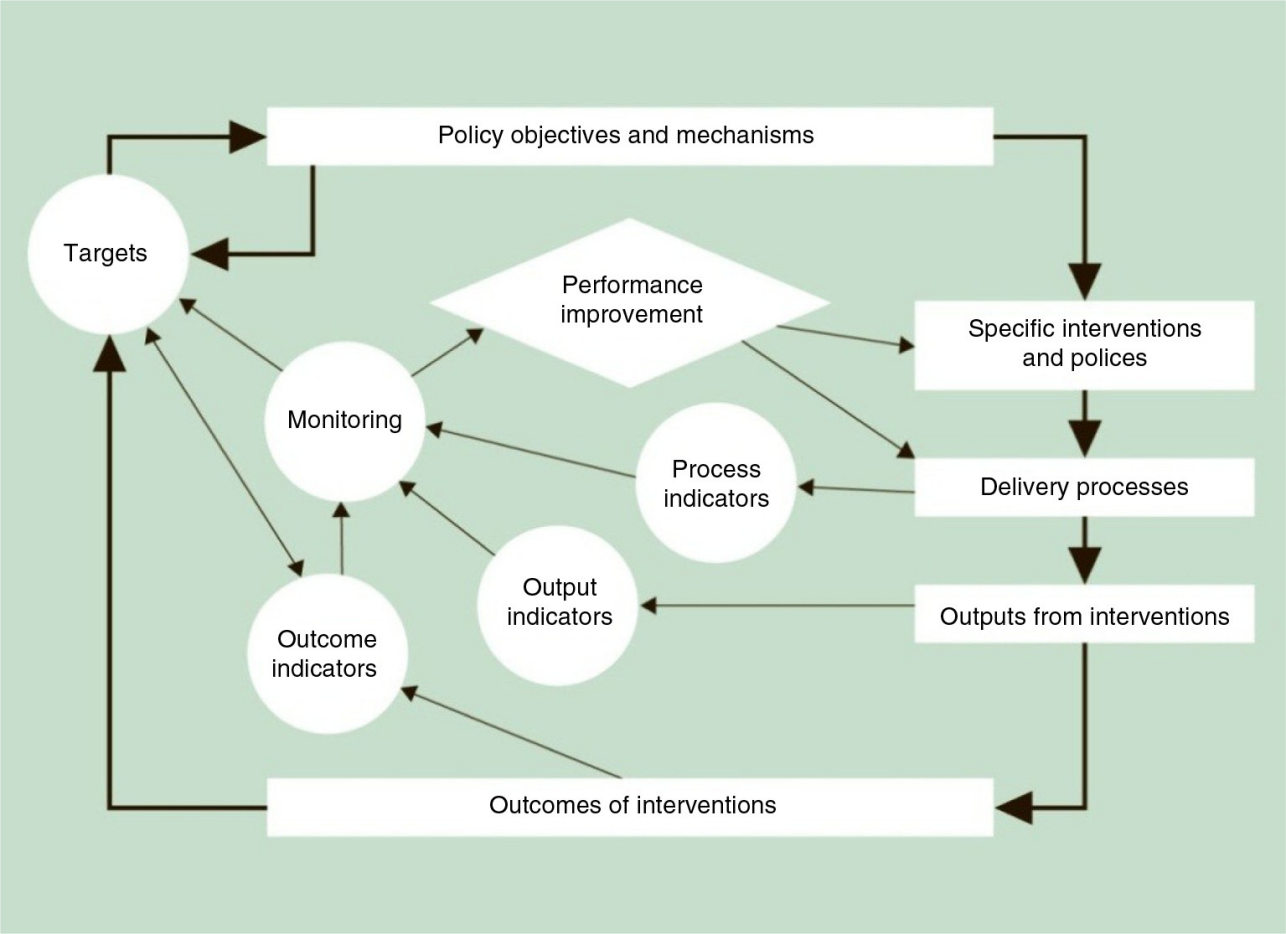
Indicator framework. Source: The Marmot Review Team (1).

Prior to 2010, the inequality strategy was monitored via the two targets (life expectancy and infant mortality) and a basket of indicators on health and its social determinants. Based on this experience, the Marmot Review proposed the monitoring framework and recommended indicators shown in Fig. 1. These recommendations were accepted and the available data were published in 2011 for every local authority. Public health responsibilities were made available on a Public Health Observatory website (11). For three of the indicators (life expectancy, health expectancy, and social inclusion), the slope of inequalities between small areas in each administrative area was also published.

### Subsequent monitoring developments in England

After 2010, the new government decided that it would not set targets – as these became regarded as being too prescriptive and introducing perverse incentives that led to negative behaviours (12). Instead, it developed three health outcome indicator frameworks for monitoring all aspects of health and social care provision – a public health outcomes framework (13), an NHS outcomes framework (14), and an adult social care outcomes framework (15). The public health outcomes framework covers five broad ‘domains’, with indicators in each domain set to monitor progress at the local level:

- Domain 1 – Health protection and resilience: protecting people from major health emergencies and serious harm to health
- Domain 2 – Tackling the wider determinants of ill health: addressing factors that affect health and well-being
- Domain 3 – Health improvement: positively promoting the adoption of ‘healthy’ lifestyles
- Domain 4 – Prevention of ill health: reducing the number of people living with preventable ill health
- Domain 5 – Healthy life expectancy and preventable mortality: preventing people from dying prematurely

Although this facilitates monitoring of inequalities of outcomes and processes between local administrative areas, it does not necessarily enable the monitoring of socio-economic inequalities within these areas. The latter requires indicators that reflect social distributions. Achievement of this objective in the indicator set is inconsistent due to the difficulties in achieving sufficient granularity in most datasets that are available on a basis that is consistent across areas.

Over time, both the indicators derived from the Marmot Review and the government's three outcome frameworks have been subject to a process of periodic amendment to take account of changes in data availability, identification of potentially useful sources of new indicators, changes in administrative rules or data definitions, new policy or political priorities, critiques of existing indicators, and new research findings.

For example, the publication *NHS Outcomes Framework: Indicators for Health Inequalities Assessment* (16) explains how the NHS outcomes framework is being used to monitor how the legal duties to reduce health inequalities are being met. It sets out the initial list of indicators for health inequalities assessment, to begin in 2015/16, and outlines the parallel process of further indicator development. Asaria et al. (17) subsequently proposed indicators for reducing inequalities in healthcare outcomes in England that could be used to monitor equity at the local and national levels and might have some applicability to universal systems internationally.

## Lessons learned

There are a number of lessons to be learned about monitoring from the developments that have taken place in England.

### Continual review

The indicators put in place at the beginning of an inequality strategy to monitor and evaluate are inevitably a relatively crude fit to the new policies and practices being implemented. Only as experience is gained of the intended and unintended consequences of new initiatives – and of the countervailing forces in society that undermine their intended effects – can more sensitive indicators be identified. At the same time, where additional or different priorities are identified in some local areas, local monitoring needs to be put in place to identify the impact of doing this (see ‘national policy and local monitoring’ section). In all these situations where required data do not exist at the start of a strategy, collection and collation of information needs to take place before the most appropriate indicators can be put in place.

For these reasons, a continual process of review is necessary. However, this recommendation does create a paradox. In many cases the trend data to adequately monitor and evaluate are not in place. Wherever possible, efforts should be made to ensure that comparisons can be made over time, for example, through parallel running or through in-depth sample studies to allow ‘bridging’ of time series over time.

### Vulnerability of administrative data

One of the major challenges to the continuity of indicator series over time lies in the need to use administrative data to monitor the implementation of policies. However, they are particularly susceptible to changes in administrative procedures or methods of collection. This has affected the continuity of indicators on determinants such as school readiness and those affected by age at leaving full-time education. To some extent, this problem can be addressed by combining survey and administrative data in an indicator, as is currently done in estimating unemployment rates. However, it is less effective in addressing more fundamental structural changes (e.g. in labour market conditions) that have underpinned changes in administrative data collections. For example, mandatory changes in either the age of ceasing participation in education or training or requirements that the young unemployed participate in short-term work activity will depress the numbers of young people ‘not in education, employment or training’, irrespective of the sustainable benefits that these changes make to long-term employment prospects (18).

### National policy and local monitoring

The emphasis on localism – on local solutions to local problems and on community empowerment – both in *Fair Society, Healthy Lives* and ‘Healthy Lives, Healthy People’ (10) – has put the spotlight on indicators available at the local and neighbourhood levels. This has inevitably meant shifting emphasis away from indicators available only at the national level – and hence those that rely on smaller in-depth surveys and cohort studies, rather than those that make use of administrative data, less timely decennial census data, or large, broad-brush multipurpose surveys. Restoring some of the balance between monitoring national and local impact would also facilitate assessing the relative contribution being made by different social determinants.

### Local data limitations – the social gradient in health and its determinants

Whereas all the indicator sets developed for monitoring cover a broad range of social determinants, only a few provide the basis for monitoring the social gradient in health and its determinants. If determinants such as preschool participation rates or environmental quality could be disaggregated by other social stratifiers, such as levels of family or local area deprivation, the social distribution of these determinants could be routinely monitored. This largely reflects the limitations of national data available locally. In general, disaggregation is only possible where small area information can be used as a proxy for individual-level socio-economic position. As remarked above, this may require making a distinction between national and local monitoring.

### Impacts of transnational and global factors

One of the limitations of the analysis in the Marmot Review was its focus on what could be achieved through action within England without attention being given to the wider contextual factors operating at transnational and global levels. These were considered in the review of health inequities in the WHO European region (19). It is arguable that monitoring in England should include contextual indicators on these wider factors.

**Fig. 2:**
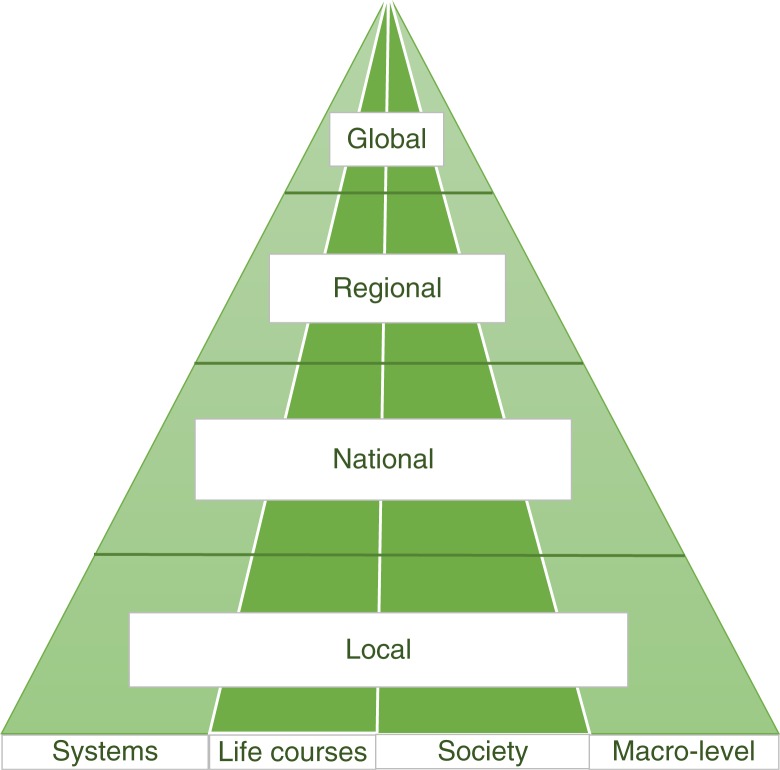
Vertical and horizontal integration of monitoring based on broad themes used in the World Health Organization European Review.

Bringing together this observation with that introduced earlier (on local monitoring) suggests that monitoring should ideally be integrated on a pyramidal basis – with high-level indicators at a global level, for example, drawing on concepts underpinning the sustainable development goals (SDGs) (20) – and then drilling down to more detailed indicators that reflect specific local priorities within the same broad social determinant themes. Figure 2 provides an idealised picture of how this monitoring across broad themes could be integrated across geographic levels.

In this context it is useful to review some of the monitoring of SDH that is undertaken internationally. The United States has, for many years, monitored disparities in healthcare and preventative health services based on ethnic origin and income, but limited routine monitoring of outcomes based on ethnic origin (21). Across the Americas, ‘Health in the Americas’ contains 5-yearly routine national-level data on determinants but more limited disaggregation within countries (22). In Europe, the European Commission monitor a broad range of determinants linked to their Europe 2020 targets at the EU, national, and regional (NUTS 2) levels – but not at more local levels (23). This monitoring includes national data on life expectancy by educational attainment for 18 countries in the European Health Programme (24).

## Conclusions

The frameworks and indicators used in England for monitoring of health inequality strategies have developed considerably since the first health inequality strategy began. Much can be learned by countries initiating or reviewing systems to monitor strategies based on addressing SDH concerning the need to develop comprehensive monitoring frameworks linked to well-defined policy objectives. The limitations identified here also provide important messages – particularly the importance of local monitoring consistent with national and global frameworks and the dependency on more disaggregated data than is generally available.

## References

[1] The Marmot Review Team (2010). Fair society, healthy lives: strategic review of health inequalities in England post-2010.

[2] Commission on Social Determinants of Health (2008). Closing the gap in a generation: health equity through action on the social determinants of health: final report of the Commission on Social Determinants of Health.

[3] Black D (1980). Inequalities in Health. Report of a research working group.

[4] Whitehead M, Townsend P, Whitehead M, Davidson N (1992). The health divide. Inequalities in health: new edition.

[5] Acheson D (1998). Inequalities in health. Report of an independent inquiry.

[6] HM Treasury/Department of Health (2002). Tackling health inequalities. 2002 Cross-cutting review.

[7] Department of Health (2003). Tackling health inequalities. A programme for action.

[8] Department of Health (2009). Tackling health inequalities: 10 years on – A review of developments in tackling health inequalities in England over the last 10 years.

[9] Bambra C, Smith KE, Garthwaite K, Joyce KE, Hunter DJ (2011). A labour of Sisyphus? Public policy and health inequalities research from the Black and Acheson Reports to the Marmot Review. J Epidemiol Community Health.

[10] Secretary of State for Health (2010). Healthy lives, healthy people: our strategy for public health in England.

[11] London Knowledge and Intelligence Team (2011). Marmot indicators for local authorities in England.

[12] Mears A (2014). Gaming and targets in the English NHS. Univ J Manage.

[13] Public Health England (2013). Public health outcomes framework.

[14] Department of Health NHS outcomes framework 2011 to 2012.

[15] Department of Health (2013). Adult social care outcomes framework 2012 to 2013.

[16] Department of Health (2015). NHS outcomes framework: indicators for health inequalities assessment.

[17] Asaria M, Ali S, Doran T, Ferguson B, Fleetcroft R, Goddard M (2016). How a universal health system reduces inequalities: lessons from England. J Epidemiol Community Health.

[18] Mirza-Davies J (2015). NEET: young people not in education, employment or training.

[19] The Institute of Health Equity (2013). Review of social determinants and the health divide in the WHO European Region: final report.

[20] Hussainpoor A, Bergen N, Magar V (2015). Monitoring inequality- an emerging priority for health post-2015. Bull World Health Organ.

[21] Agency for Healthcare Research and Quality (2014). National healthcare quality and disparities report.

[22] Pan American Health Organization Health in the Americas 2012.

[23] Eurostat Europe 2020 Indicators.

[24] Eurostat (2015). Life expectancy by age, sex and educational attainment.

